# A Preliminary Investigation of the Jack-Bean Urease Inhibition by Randomly Selected Traditionally Used Herbal Medicine

**Published:** 2012

**Authors:** Mahmood Biglar, Khadijeh Soltani, Farzaneh Nabati, Roya Bazl, Faraz Mojab, Massoud Amanlou

**Affiliations:** a *Department of Medicinal Chemistry, Faculty of Pharmacy and Medicinal Plants Research Center, Tehran University of Medical Sciences, Tehran, Iran.*; b *Department of Pharmacognosy, School of Pharmacy, Shahid Beheshti University of Medical Sciences, Tehran, Iran.*

**Keywords:** Herbal extract, Urease, Inhibitor, Indophenol method, Lead discovery

## Abstract

*Helicobacter pylori *(*H. pylori*) infection leads to different clinical and pathological outcomes in humans, including chronic gastritis, peptic ulcer disease and gastric neoplasia and even gastric cancer and its eradiation dependst upon multi-drug therapy. The most effective therapy is still unknown and prompts people to make great efforts to find better and more modern natural or synthetic anti-*H. pylori *agents. In this report 21 randomly selected herbal methanolic extracts were evaluated for their effect on inhibition of Jack-bean urease using the indophenol method as described by Weatherburn. The inhibition potency was measured by UV spectroscopy technique at 630 nm which attributes to released ammonium. Among these extracts, five showed potent inhibitory activities with IC_50_ ranges of 18-35 μg/mL. These plants are *Matricaria disciforme *(IC_50_:35 μg/mL), *Nasturtium officinale *(IC_50_:18 μg/mL), *Punica granatum *(IC_50_:30 μg/mL), *Camelia sinensis *(IC_50_:35 μg/mL), *Citrus aurantifolia *(IC_50_:28 μg/mL).

## Introduction

Many hundreds of plants worldwide are used in traditional medicine as treatments for different kinds of diseases including bacterial infections and gastrointestinal disorders. Among these bacteria, *H. pylori*, a Gram-negative pathogenic bacterium which specifically colonizes the human gastric mucosa, has been regarded as a primary causative agent of chronic gastritis and peptic ulcer diseases including mucosa-associated lymphoid tissue lymphoma (1). Conventional multiple drug therapy in management of *H. pylori *infection usually provide effective therapy but there is an increasing problem of antibiotic resistance, side effects and significant cost of therapy which associate with these kinds of drugs (2-4).

While *H. pylori *is acid sensitive and only replicates at pH of 7-8, it survives in the stomach under highly acidic conditions (5-7) urease activity in bacteria is believed to be essential for the colonization of and survival of *H. pylori *at very acidic pH (8, 9). Thus virulence of *H. pylori *could be controlled using chemicals that inhibit urease activity.

Ureases (E.C 3.5.1.5), the first enzyme crystallized from Jack been (*Canavalia ensiformis*) was shown to contain nickel ions (10) which rapidly catalyzes the hydrolysis of urea to form ammonia and carbon dioxide (11) have been shown to be an important virulence determinant in the pathogenesis of many clinical conditions, which is detrimental for human and animal health as well as for agriculture (12). The product, ammonia, of such decomposing reactions diffuses across the cytoplasmic membrane, buffering the periplasmic space and allows growth in the presence of extracellular gastric acid (13), and responsible for negative effects of urease activity in human health (14), such as causing peptic ulcers, stomach cancer, etc. Besides, in agriculture the efficiency of soil nitrogen fertilization with urea decreases due to ammonia volatilization and root damage caused by soil pH increase (15).

Then, it’s interesting to control the activity of urease through the use of its inhibitors in order to counteract these negative effects in medicine, environmental and agronomic. Many urease inhibitors have been described in the past decades, such as phosphorodiamidates (16), *α-*hydroxyketones (17), Polyhalogenated benzo- and naphthoquinones (18) and imidazoles such as proton pump inhibitors of lansoprazole, rabeprazole and omeprazole (19).

Natural urease inhibitors from *Euphorbia decipiens *(20) and sulfated polysaccharide found mainly in various species of brown seaweed (fucoidan compounds) had been reported previously (21). The use of some chemical or herbal compounds were banned in vivo or entering clinical trials because of their toxicity, chemical or physical instability or low bioavailability (22).

Thus, seeking novel and efficacious urease inhibitors with good bioavailability and low toxicity are significative especially in low income countries with high infection rate of *H. pylori *is desirable. This report is focused on seeking for novel natural urease inhibitors from herbal sources that can be used directly or as a lead compounds in management of *H. pylori *infection.

## Experimental

*Plant extraction and preparation of extracts*

Twenty one medicinal plants which are listed in Table 1, are obtained randomly from local herbal market in September 2010, Tehran and identified by one of authors, Dr. F. Mojab and were evaluated against Jack-bean urease. Each plant sample was individually powdered and 1 g was extracted by maceration method using aqueous methanol (10 mL; 50:50 v/v) as solvent for 24 h. Each extract was filtered, concentrated under reduced pressure to dryness and stored at 0°C until time of analysis. The percentage of inhibition at 1000 μg/mL concentration of extracts, dissolved in same solvent was accurately defined.

**Table 1:** Name of the plants, part used in traditional medicine and percent of inhibition of urease enzyme in presence of 1 mg/mL of each herbal extract

	**Scientific name**	**Common name in English**	**Common name in Persian**	**Part used**	Percent of inhibition (%)*
**1**	*Alhagi maurorum*	Camel thorn	Khar-e shotor	resin	32.0 ± 1.1
**2**	*Boswellia carterii*	Frankincense	Kondor	resin	11.3 ± 1.9
**3**	*Camelia sinensis*	Tea shrub	Chai	leaf	95.4 ± 2.40
**4**	*Cerasus avium*	Cherry tail	Dom-e gilas	tail	26.6 ± 2.90
**5**	*Citrus aurantifolia*	Basra lime	Limu Omani	fruit	97.6 ± 0.78
**6**	*Citrullus colocynthis*	Bitter apple	Hanzal	Fruit	70.1 ± 0.91
**7**	*Cotoneaster nummularia*	Pockspary manna	Shirkhesht	resin	10.3 ± 0.47
**8**	*Fraxinus velutina*	Velvet Ash	Zaban-e ghonjeshk	leaf	16.4 ± 0.55
**9**	*Laurus nobilis*	Grecian laurel	Bargh-e bu	leaf	68.8 ± 1.63
**10**	*Matricaria recutita*	Chamomile	Babun-e shirazi	flower	88.8 ± 0.99
**11**	*Nardostachys jatamansi*	Spikenard	Sonboletib	rhizomes	0
**12**	*Nasturtium officinale*	Watercresses	Bolagooti	leaf	99.1 ± 1.78
**13**	*Nepeta bracteata*	Catmint	Zufa	flower	21.4 ± 2.24
**14**	*Nepeta menthoids*	French lavender	Stoqodus	branch	26.6 ± 1.43
**15**	*Physalis alkekengi*	Winter cherry	Arusak-eposht-e pardeh	fruit	72.2 ± 1.88
**16**	*Polygonum aviculare*	Seresh	Alafe haft band	leaf	62.5 ± 3.10
**17**	*Punica granatum*	pomegranate	Golnar	flower	96.7 ± 2.55
**18**	*Salvia officinalis*	Sage	Maryam Goli	leaf	71.3 ± 0.22
**19**	*Sambucus nigra*	Black Elder	Agti	leaf	41.0 ± 1.28
**20**	*Trachyspermum copticum*	Ajwain	Zenyan	seed	38.8 ± 1.67
**21**	*Zea mays*	Corn Crest	Kakol-e dhorrat	noodle	22.2 ± 1.47

Values are expressed as mean ± SD of 3 experiments

*Chemicals*

All the chemicals used were of analytical grade from Merck Co., Germany. All aqueous solutions were prepared in MilliQ (Millipore, USA) water. Jack-bean urease was obtained from Merck (5 units/mg).

*Urease inhibition activity assay*

For urease inhibition assays after addition of 10 mL of phosphate buffer to accurately weight of enzyme, sonication was performed for 60s, followed by centrifugation and evaluating absorbance of upper solution in λ = 280 nm which is attributed to enzyme. By using the following equation A = ε*bc *where *c *is the concentration of solution (mol/L), *b *is the length of the UV cell and ε represents molar absorptivity in the specific wavelength, we can calculate the concentration of initially urease solution. After proper dilution, the concentration of enzyme solution adjusts at 2 mg/mL.

The assay mixture, containing 100 μL (2 mg/mL) of Jack-bean urease and 100 μL of the test compound with 0.2 mL of 100 mM phosphate buffer pH 6.8 containing 25 mM urea was pre-incubated for 30 min in water bath at 37°C. The urease reaction was stopped after 30 min incubation with 600 μL of 4% H_2_SO_4_ acid. Enzyme inhibition activity performed by Berthelot alkaline phenol–hypochlorite method to examine the efficiency of adsorptive immobilization. This method is based on the released ammonia (NH_3_) which reacts with hypochlorite (OCl^−^) to form a monochloramine (23). This product then reacts with phenol to form blue-colored indophenols whose absorbance is measured at 625 nm.

The liberated ammonia was estimated using 500 μL of solution A (contained 5.0 g phenol and 25 mg of sodium nitroprusside) and 500 μL of solution B (contained of 2.5 g sodium hydroxide and 4.2 mL of sodium hypochlorite in 500 mL of distilled water) at 37°C for 30 min and the absorbance was measured at 625 nm against the control. All reactions were performed in triplicate in a final volume of 1 mL. Percentage of inhibitions were calculated using the formula (100 - (OD sample / OD control) × 100).

The concentration of compounds that inhibited the hydrolysis of substrate by 50% (IC_50_) was determined through monitoring the inhibition effect of various concentrations of extracts in the assay. The IC_50_ values were then calculated using above mentioned formula in the previous section.

## Results and Discussion

As evidence in beneficial effects of medicinal plants traditionally used to manage different disorders, twenty one samples, are available from local herbal and medicinal plants shop were examined against Jack-bean urease by Berthelot alkaline phenol-hypochlorite method and results revealed varied inhibitory activities (Table 1). Five extracts which showed maximum inhibitory effect (≥ 90 % of enzyme inhibition) were selected and further studied for IC_50_ determination by UV-spectroscopy technique; the relevant dara is presented in Table 2.

**Table 2:** The IC_50_ and the percent of enzyme inhibition in the presence of plants extract at concentration of 1 mg/mL

**Plant name**	**IC**_50_ ** (μg/mL)**	**Percent of Inhibition (%)**
*Camelia sinensis*	35 ± 1.9	95.38 ± 2.40
*Citrus aurantifolia*	28 ± 0.6	97.64 ± 0.78
*Matricaria recutita*	37 ± 1.6	88.88 ± 0.99
*Nasturtium officinale*	18 ± 1.4	99.13 ± 1.78
*Punica granatum*	30 ± 1.2	96.75 ± 2.55

Preincubation time was 0.5 h. IC_50_ vales were calculated from urease inhibition rate of six doses, 10, 20, 40, 60 and 80 μg/mL in triplet

**Figure 1:**
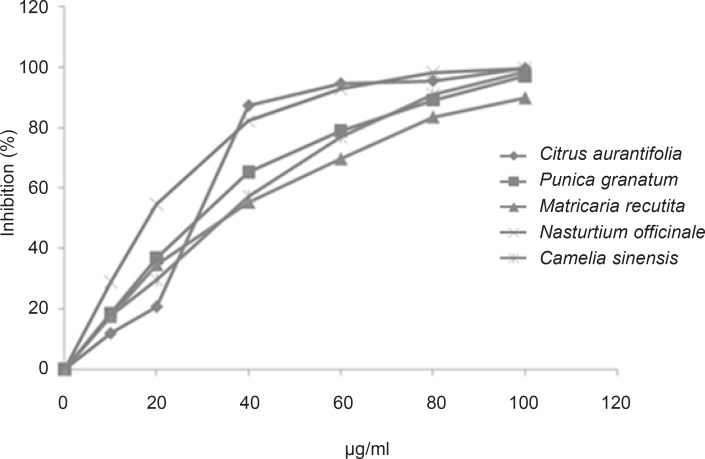
Inhibition profile of five selected extract against Jack-been urease activity by indophenol method

As shown in Figure 1, concentration-dependent activities against Jack-bean urease were observed between selected extracts and inhibitory effect increased together with increasing the concentration of each plant’s extract in the range of (0-100 μg/mL).

As shown in Table 2, the inhibitory activities of five selected extracts were found to be the most potent inhibitors are: *Camelia sinensis *(IC_50_ = 35 μg/mL), *Citrus aurantifolia *(IC_50_ = 28 μg/ml), *Nasturtium officinale *(IC_50_ = 18 μg/mL), *Punica granatum *(IC_50_ = 30 μg/mL) and *Nasturtium officinale *(IC_50_ = 35 μg/mL).

Medicinal plants serve as a useful sourcees of novel drugs (24). In developing countries, since the application of antibiotics is still under a poor management as a whole, there is a growing need for finding new medicinal plants especially anti-*H. pylori *agents that can help eradicate the invasion and presence of survived *H. pylori *strains to avoid relapse of gastric ulcer. In this regard, the literature has reported extracts of certain plants such as cashew apple (25), cinnamon (26), and Chinese tea (27) inhibit growth of *H. pylori *and some urease inhibitory activity.

According to reported investigations on *Camelia sinensis *(Green tea) its *H. pylori*’s urease inhibition has been proven (28). Achieving IC_50_ equal to 35 μg/mL for *C. sinensis *in this study which is varied from previous researches (IC_50_ = 13 μg/mL), is probably due to the diversity of growth, harvesting and extraction methods. Regarding the excess amount of flavon, flavol, catechin epigallocatechin gallate, gallocatechin gallate, gallocatechin, and many other flavonoids in *C. sinensis *(28)*, *we can conclude that urease inhibitory activity of black tea (this study) is attributed to the similar substances found in green tea extract in different amounts. The chemical constituents and functional groups of these compounds play important role in inhibition of urease. The functional groups such as hydroxyl and ketones which are linked with aromatic rings can interact with Ni ions in active site of enzyme, resulting in its inhibition.

The Key lime (*Citrus aurantifolia*; Omani lime) is a citrus species with a globose fruit, 2.5-5 cm in diameter (1–2 in) that is yellow when ripe but usually picked green commercially. It is smaller, seedier, has a higher acidity, a stronger aroma, and a thinner rind than that of the Persian lime (*Citrus x latifolia*). It is valued for its unique flavor compared to other limes, with the key lime usually having a more tart and bitter flavor. In Malaya, the juice is taken as a tonic and to relieve stomach ailments and it is given as a vermifuge in combination with oil. In India, the pickled fruit is eaten to relieve indigestion. It have been used as an antiseptic, tonic, an antiscorbutic, an astringent, and as a diuretic in liver ailments, a digestive stimulant, a remedy for intestinal hemorrhage and hemorrhoids, and as a disinfectant for all kinds of ulcers when applied in a poultice (29-33). In Iran dried fruits are usually consumed as vegetable and prevent indigestion.

The chemical composition of *C. aurantifolia *is well known and limonene, *γ*-terpinene, terpinolene and *α*-terpineol present in different amounts (32). Antibacterial activity of essential oil of lime is related to its composition (31) but none of those compounds could inhibit urase enzyme. However urease inhibitory of *C. aurantifolia *was found to be (IC_50_ = 28 μg/mL). The methanolic extract of dried fruits may contain new substance which is not reported in literature and need more investigation on its isolation and identification.

Another plant which shows high inhibitory effect on urease is *Matricaria recutita *(*Chamomile*). Previously, it was shown that the *Chamomilla recutita *(*M. recutita*) oil extract is rich in fatty acids, coumarins, terpenes, spiroethers and flavonoids contributing to its medicinal properties (34, 35). Additionally, *Roman chamomile *sample showed high antimicrobial activity against all strains of tested microbes Gram-negative bacteria (*Escherichia coli, Pseudomonas aeruginosa, Proteus vulgaris, Klebsiella pneumoniae and Salmonella sp.*) (36, 37).

The chamomile oil inhibited the *H. pylori’s *growth in very low concentrations of 0.0075% (v/v). The reported MIC_50_ and MIC_90_ of *Chamomilla recutita *(*M. recutita*) oil extract for *H. pylori *were 62.5 mg/mL and 125.0 mg/mL, respectively. In addition, it was found that urease production of *H. pylori *was inhibited by the *M. recutita *oil extract (35). This finding was of particular interest, since urease activity is critical for the survival of this microorganism in the stomach.

It was shown that the *M. recutita *oil extract influenced the morphological and fermentative properties of *H. pylori*. Thus, it is possible that these compounds (fatty acids, coumarins, terpenes, spiroethers and flavonoids) could be responsible for the morphological and fermentative changes and subsequent anti-*H. pylori *activity of the *M. recutita ita *oil extract. However, a 50 % aqueous methanol extract from *M. recutita *flowers was also found to be inhibitory against urease, which corroborates previously reported results (35). Both *M. recutita *oil extract or its methanloic extract may be useful as additional remedy in the complex treatment of stomach ulcers and duodenal intestinal diseases which are subjected to *H. pylori*, especially for patients with allergic responses to antibacterial drugs (35).

Watercresses (*Nasturtium officinale*) are fast-growing, aquatic or semi-aquatic, perennial plants native from Europe to central Asia, and one of the oldest known leaf vegetables consumed by human beings. These plants are members of the family Brassicaceae or cabbage family, botanically related to garden cress and mustard with a peppery, tangy flavor due to high contents of phenethyl isothiocyanate (38). Many benefits from eating watercress are claimed, such as that it acts as a stimulant, a source of phytochemicals and antioxidants, an anti-microbial, a diuretic, an expectorant, and a digestive aid (38-40). The phenethyl isothiocyanate content of watercress inhibits hypoxia-inducible factors which can inhibit angiogenesis in lung cancer (41). The strong urease activity (IC_50_ =18 μg/mL) reported in this study among all other extracts, which reported for the first time, is concurrent with its daily usage as digestive aid in people whom suffering gastric upset. Phenethyl isothiocyanate may interact with urease Ni ions and inhibit its catalytic activity. More investigationa are needed to confirm this hypothesis.

Another plant which shows positive effect on urease inhibition is *Punica granatum *(Pomegranate**)***. *Previous studies on pomegranate demonstrated its medicinal usage against *salmonella *(42). Anti-*helicobacter pylori *activity of aqueous and ethanolic extract of *punica granatum *pericarps are recently reported (43). The minimal inhibitory concentration (MIC) value was reported to be 0.78-6.25 mg/ mL, but the mechanism of this inhibition was not clearly investigated.

The medicinal plant possesses a high amount of tannin (25%). The antimicrobial properties of this substance were well established. Polar fraction of *P. granatum *was reported to contain ellagitannin and punicalagin (43). The strong urease inhibitory activity of *P. granatum *(IC_50_ 30 μg/mL) reported here revealed that antibacterial properties of *P. granatum *against different strands of *H. pylori *could be at least partially due to inhibition of urease by interaction of ellagitannin and punicalagin with active site of enzyme or modulating its activity by aggregation properties of tannin.

Medicinal plants, traditional medicinal and other natural sources are still good source for lead discovery. The results of this study revealed that random screening of medicinal plants could lead to introducing new candidate for further studies which, in the end, can help and enhance human health.
